# Patient and Parent Knowledge, Understanding, and Concerns After a New Diagnosis of Ehlers Danlos Syndrome

**DOI:** 10.21203/rs.3.rs-4433259/v1

**Published:** 2024-06-10

**Authors:** Jordan T. Jones, Lora L. Black, William R. Black

**Affiliations:** Children’s Mercy Kansas City; The Ohio State University; Abigail Wexner Research Institute at Nationwide Children’s Hospital

**Keywords:** Ehlers Danlos syndrome, Education, Concerns, Barriers, Patient and Parent Knowledge

## Abstract

**Introduction:**

After diagnosis of Ehlers Danlos Syndrome (EDS), it is unclear what information patients and parents need and understand about EDS. The objective of this study is to characterize patient and parent knowledge and concerns about EDS after a diagnosis of EDS is made to determine patient and parent concerns and identify barriers that cause discomfort with the diagnosis.

**Methods:**

A convenience sample of patient and parent dyads were recruited after new diagnosis of EDS. Patients and parents completed questionnaires that assessed knowledge, comfort, and barriers of EDS before and after diagnosis, EDS education materials accessed, and additional clinical needs and concerns.

**Results:**

Seventy-two dyads completed the survey.

**Conclusion:**

Many respondents actively seek information on the diagnosis and management of EDS. Parents and patients look for information about EDS differently. Parents have more concerns after diagnosis and both want well-constructed, empirically supported educational materials delivered via multiple modalities, which makes clinical guidelines more essential.

## Introduction

The Ehlers-Danlos Syndromes (EDS) are a group of heritable connective tissue disorders with overlapping phenotypic features which include joint hypermobility, skin hyperextensibility, and tissue fragility^1,2^. There are 13 recognized distinct clinical subtypes^1^ with different clinical features and genetic abnormalities. Definitive diagnosis relies on molecular confirmation with all subtypes except for hypermobile EDS, which is based on a clinical criteria^1^, though a genetic basis is suspected for hypermobile EDS^3^. Clinical sequalae for patients with EDS is often complex and spans across multiple body systems^4,5,6^ and is commonly associated with chronic pain^7^, anxiety, and depression^8^. Care for patients with EDS is multifaceted due to the array of symptoms and body systems involved in the disorders, and due to the multisystem involvement, diagnosis of EDS may be delayed or misdiagnosed^9^.

EDS is likely the most common systemic inherited connective tissue disorder in humans, with a minimum prevalence estimated at 1 in 5000; however, the prevalence is likely much higher^10^. This diagnostic gap may be due to several reasons, which includes misdiagnosis, too few providers trained in the diagnosis and management of EDS, and lack of confidence in the diagnosis and management of EDS^11,12^. Previous studies have shown that many providers are uncomfortable with diagnosis, care, and management of patients with EDS and note the existence of many barriers that prevent adequate care^11,12^. For this reason, multidisciplinary teams with EDS expertise have been developed^13,14^ to aid in diagnosis, care and management with appropriate referrals and education to prevent new or worse symptoms that may lead to poor quality of life (QoL). Despite the increase in multidisciplinary EDS clinics there is still a limited number of medical providers familiar with EDS to service the population needs, which further contributes to long delays in diagnosis and treatment and may limit the transmission of empirical medical information to patients and families.

The diagnostic journey for patients with EDS is rarely straightforward and often burdensome^15^. It regularly involves referrals to many specialists, but patients report being provided with few definitive answers, which leads to negative outcomes such as mistrust in medical providers^16^. After diagnosis, patients and families are typically provided EDS focused education on diagnosis and symptoms^13^, however, there are no standard recommendations on when EDS education should occur or what it should include. The timeframe between diagnosis and initiation of active management may be a “window of opportunity” to appropriately educate families, identify age-specific needs and concerns, build trust and confidence, and develop age-appropriate care strategies that could lead to improved outcomes and QoL. It is vital to understand what patients and families initially understand about the disease process, what general or age-specific needs they require due to the disease, and what questions they would most like to have answered as part of their care to better tailor the education provided to patients and families.

The objective of this study was to characterize patient and family knowledge and awareness of EDS after a diagnosis is made, determine patient and family concerns about the diagnosis and care, and identify barriers that cause discomfort for patients and families.

## Methods and Materials

This work was conducted in accordance with the Declaration of Helsinki, and local institutional review board (IRB) approval was obtained. Prior to participation, the study was explained to each study participant and their parent/legal guardian. Written informed consent and assent were waived by IRB. A convenience sample of patient and parent dyads were recruited from a multidisciplinary EDS clinic in sequential order over a 9-month period between April and December 2023, provided the eligibility criteria were met. Patients between the ages of 8 and 22 years were included if they were diagnosed with EDS using the 2017 International Classification of EDS^1^ within the previous three months and had a parent/legal guardian that would agree to participate in the study. Patients were excluded if they did not have a recent diagnosis of EDS or did not have a parent/legal guardian that was willing to participate. Patients and a parent/legal guardian simultaneously completed an electronic survey via REDCap electronic data capture that consisted of 31 questions for the parent/legal guardian and 24 questions for the patient participant. Questions assessed knowledge and awareness of EDS before and after diagnosis, comfort with the diagnosis of EDS, barriers that prevented comfort with EDS diagnosis, EDS education materials accessed, and additional clinical needs and concerns. Demographic information was collected from all participants. The survey utilized Likert-scale^17^ responses on a 5-point scale (“not at all comfortable” to “completely comfortable”), questions with the ability for selecting multiple responses (e.g., “choose all that apply”). The electronic survey was developed for this study and not previously validated.

## Statistical Analysis

Descriptive statistics were conducted. Binary and categorical variables were summarized by frequency and percentage. All statistical analysis was completed using SPSS statistics 24 software.

## Results

### Participant demographics

Overall, 94 parent/legal guardian and patient dyads were screened and 76 were eligible for the study, while 74 were approached and enrolled. Responses from the 74 patient and parent/legal guardian dyads were analyzed, while two patient and parent/legal guardian dyads responses were incomplete and not included in the final analysis. Of the 72 parent/legal guardian responses, 96% were a parent with the majority (57%) between the age of 40 and 49 years. Most were married (81%), Caucasian (85%), females (93%) that reported 4 to 6 total people in the household (65%). Of the parent/legal guardians 20% had a personal diagnosis of EDS and 30% suspected they had EDS but had not been diagnosed. Half (51%) of the parent/legal guardians reported 1 to 3 family members with EDS. Most were employed full-time (68%), with some college, but no degree (31%) and have an average household income of greater than $100,000 per year (33%). Of the 72 patient responses, most were between the age of 16 and 18 (51%). Most were female at birth (81%) and 13% were gender diverse (Table 1.).

### Familiarity with EDS knowledge and barriers to knowledge acquisition

Of the surveys analyzed, 57% of parents and patients had heard of EDS prior to their diagnosis. Of those that had heard of EDS, most parents (26%) heard of EDS from a medical provider or family member, while most patients (22%) heard about EDS from a parent or other diagnosed patient. More patients (18%) than parents (10%) heard about EDS from social media. Most parents (67%) and about half the patients (49%) were “somewhat comfortable” with their understanding of EDS. The biggest barriers to parental comfort with EDS knowledge were a lack of knowledge (47%), educational materials about EDS (42%), and confidence with providers knowledge of EDS (42%), while the biggest barriers to patient comfort with EDS knowledge was lack of knowledge (57%) and educational materials (32%) (**Table 2**).

### Familiarity with EDS symptoms and barriers to comfort with symptoms

Most parents (72%) reported being “somewhat comfortable” with identification and management of EDS symptoms, while 62% of patients reported being “somewhat comfortable”. Participants were also asked what barriers prevent their comfort with management of EDS and the associated symptoms. Parents noted their biggest barrier was uncertainty about which symptoms were associated with EDS and which were not (51%), followed by lack of confidence with provider knowledge of EDS (45%), and lack of education materials (38%). Patients noted their biggest barrier to comfort with symptoms associated with EDS were lack of knowledge (37%), lack of education materials about EDS (32%), and uncertainty about which symptoms were associated with EDS and which were not (32%) (**Table 2**).

### Educational Materials

Prior to a diagnosis of EDS, 43% of parents and 30% of the patients accessed online educational materials about EDS, whereas after the diagnosis of EDS, 76% of parents and 43% of patients accessed online educational materials. After patients were diagnosed with EDS, 58% of parents and 77% of patients reported they were given educational materials about EDS at their medical visit.

### Family EDS Concerns

**Figure 1:**
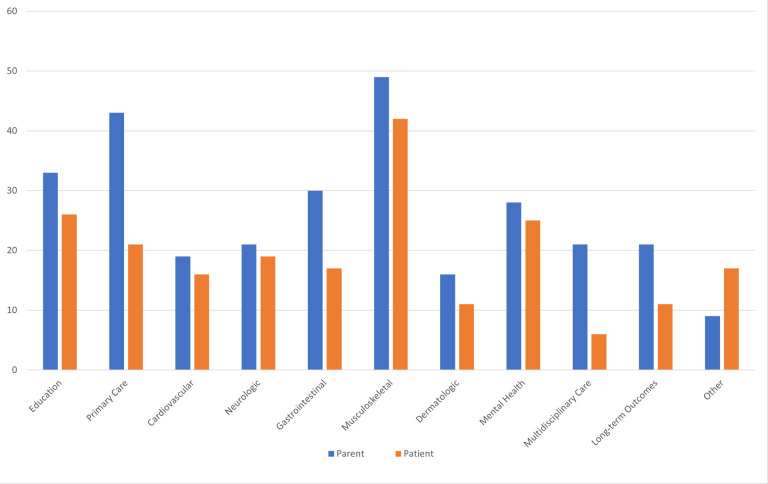
Concerns and Clinical Needs of Parent and Patient Before Diagnosis of EDS

When asked what concerns and clinical needs were present prior to EDS diagnosis, parents voiced concern about musculoskeletal care (66%), primary care for EDS (58%), education about EDS (45%), and gastrointestinal care (41%). After diagnosis, parents continued to have concerns about musculoskeletal care (66%), education about EDS (60%), primary care for EDS (55%), and gastrointestinal care (45%). Additional concerns and clinical needs reported by parents included mental health (46%) and multidisciplinary care (46%). Among patients, concerns and clinical needs prior to diagnosis included concerns about musculoskeletal care (57%), education about EDS (35%), and mental health care (34%), while after diagnosis of EDS, patients had concerns about musculoskeletal care (54%), education about EDS (47%), and long-term outcomes (37%) (Figs. 1. & 2.)

## Discussion

This is the first survey of patients and families to assess knowledge of EDS within 3 months after a diagnosis of EDS is made to determine patient and family concerns about diagnosis and care as well as identify causes of discomfort with EDS for patients and families. About half of the surveyed patients and parents had a general familiarity with EDS prior to the diagnosis. Among parents, most of their familiarity was obtained from medical providers, while a higher proportion of patients obtained additional information from social media. As expected, the percentage of both parents and patients that accessed online education about EDS was higher after the diagnosis of EDS compared to pre diagnosis. While it would be expected that all patients and families receive education about EDS after the diagnosis, 42% of parents and 33% of patients reported that they were either not provided or were unsure if they were provided with such education. Our findings about exposure to EDS education are especially important given that many patients and parents felt that a lack of educational materials were a barrier to their knowledge and comfort with EDS. Participants’ perceptions about their limited knowledge about EDS and access to educational materials may be reflective of either a dearth of available physical materials or a feeling that the available materials lacked specifics about the condition. However, this is clearly an area of opportunity to develop and improve educational offerings for patients and families.

While educational materials are commonly distributed or referenced after a medical diagnosis is made, there may be additional challenges to overcome and opportunities for improvement in post-EDS diagnosis education to improve the successful transmission of EDS knowledge to all patients and parents. Although not directly assessed with the current survey, patients and parents may be overwhelmed with a new diagnosis and all the potential comorbidities associated with EDS^18^ so clarity must be established for the patient and parent about the specific EDS subtype. The EDS subtypes could be an area of possible confusion and they may not be aware of the multiple subtypes or the subtype they have been diagnosed with specifically. Further confusion may lie in the large variability in severity among and within the various EDS subtypes^19^. Additional confusion or uncertainty about the diagnosis of EDS may exist for patients and families due to either a lack of genetic testing or negative results from genetic tests that are performed. This may be most problematic for those with the hypermobile subtype as there is no definitive genetic test and diagnosis must rely solely on clinical criteria^1^. The lack of genetic testing may confuse patients and parents if they had expectations that genetic testing would be required for a diagnosis of a heritable connective tissue disease. Additionally, when newer diagnostic criteria and frameworks^20^ are introduced, it can cause increased anxiety and confusion for patients and parents as a family member may have a diagnosis removed or changed. A focus on EDS subtype-specific educational materials may be warranted to overcome these challenges. This may provide an opportunity to improve how EDS information is provided, such as routine post-visit consultations to provide additional information, or other varied modalities such as hand-outs with visuals, online resources, and online patient advocacy groups.

Roughly half of respondents indicated no prior knowledge of EDS. Even if these families were provided with appropriate EDS education at diagnosis, the provision of medical education does not necessarily equate to comprehension. Given the amount of information that can be provided, and often lengthy clinic visits, patients and their families may become fatigued and overwhelmed by the amount of information. While clear educational materials are one strategy to consider, additional planned follow-up clinic visits or communications may provide the opportunity for families to seek clarifications, ask additional questions, and review their understanding from their clinic visit. The use of education coordinators for newly diagnosed patients has been utilized successfully in pediatric diabetes to improve adherence and disease outcomes and are part of recommended standard care^21^. A similar role may provide useful in the education and management of EDS.

While patients and parents were generally comfortable with their knowledge of EDS, symptoms, and management, there were other areas of noted concern. One such area was in the differentiation of symptoms related to EDS versus other health conditions. This is a difficult area to tackle as there are many symptoms and conditions that overlap with EDS. Medical providers need to educate families on common symptoms that may be experienced and be ready to investigate symptoms that are more ominous to ensure optimal health. This paradigm may be difficult for providers, especially as many are not comfortable with EDS or management of EDS^11^. Further, in this survey parents noted concerns and barriers related to their confidence in their providers’ knowledge of EDS. When patients or parents are uncomfortable with their provider or medical care, it may lead to lack of faith in the medical establishment and result in patients and parents to look elsewhere for healthcare guidance^16^. There is an urgent need for clinical guidelines for the treatment and management of patients with EDS to increase awareness of both common and rare comorbidities and symptoms so that appropriate evaluations and tests can be conducted in a timely manner, patients can be monitored for future comorbidities and problematic symptoms, and to provide a clear framework for initial symptom management, which could potentially improve patient confidence in the recommendations that are provided.

Understandably, we see that after diagnosis of EDS, parental concerns rise in all categories as parents learn more about EDS and possible comorbidities. For patients, EDS concerns before and after diagnosis are mostly the same except for increased concern about long-term outcomes after diagnosis. This may be due to patients’ developmental stage prior to diagnosis where their long-term health may not be as important to them as other areas of life. Their concerns may increase with age as new symptoms arise and efforts are made to better understand new symptoms in context of their overall EDS diagnosis. Further, patients may begin to have more concerns or questions as they transition into adult care and are more responsible for managing their own health. Currently, little is known about the needs of pediatric patients with EDS as they transition to adulthood, nor are there clear recommendations on transition pathways for these patients.

The results of this survey show that there is a continued need to develop educational interventions for patients and families, especially shortly after diagnosis of EDS. Differences between how adolescents and their parents have obtained information speaks to the need to develop different educational programs and strategies for parents compared to their child or adolescent. Given the role that patient networks can play in the dissemination of medical information, there may be a role for social-media specific EDS knowledge dissemination directed towards youths with EDS. Educational efforts aimed at this window of time can help improve knowledge and understanding of EDS earlier in their medical journey and help guide long-term management of symptoms and ultimately improve outcomes and QoL^22^. As educational materials and interventions are developed and distributed, for both patients, parents, and for medical providers^11^, it will be important to elicit feedback from patients, their families, and medical providers so that the information provided addresses the knowledge gaps and concerns most appropriate to the patient or provider population, which should build confidence in their ability to effectively manage the condition.

Our study has several limitations. The survey was completed locally and thus the findings may not be generalizable to larger groups in different geographical regions. Additionally, our institution has a multidisciplinary EDS clinic that serves the region, and our survey participants may have more exposure to and awareness of EDS than the larger patient population – though, this study may overestimate family knowledge of and comfort with EDS. In general, we expect the barriers reported in this study to be present and potentially more profound in areas without a multidisciplinary EDS clinic. Recall bias may also be present as participants were asked to recall concerns prior to the diagnosis of EDS when they completed the survey, however, the timeframe between diagnosis and survey was three months or less and the results of this study are consistent with the expectation that concerns could increase as more knowledge is gained. Further, this study was conducted prior to the publication of the 2023 pediatric joint hypermobility framework^20^ and may not accurately represent the concerns of patients and parents related to the updated diagnostic criteria in the pediatric population. Additionally, sample size and power analysis were not performed for this study, and the electronic survey has not been previously validated.

## Conclusions

This is the first study to assess patient and family knowledge of EDS after a diagnosis, determine concerns about the diagnosis and management, and identify barriers that cause discomfort for both patients and their parents. There were many concerns noted by patients and parents prior to the diagnosis of EDS and more concerns for parents after their child’s diagnosis. Additionally, a lack of knowledge and educational materials were the biggest barriers to comfort with EDS for many patients and parents, while uncertainty about what symptoms are related to EDS was the biggest barrier to comfort for parents. More research is needed to confirm these findings as well as develop and pilot optimal age-appropriate educational modalities for patients and their parents.

## Figures and Tables

**Figure 2:**
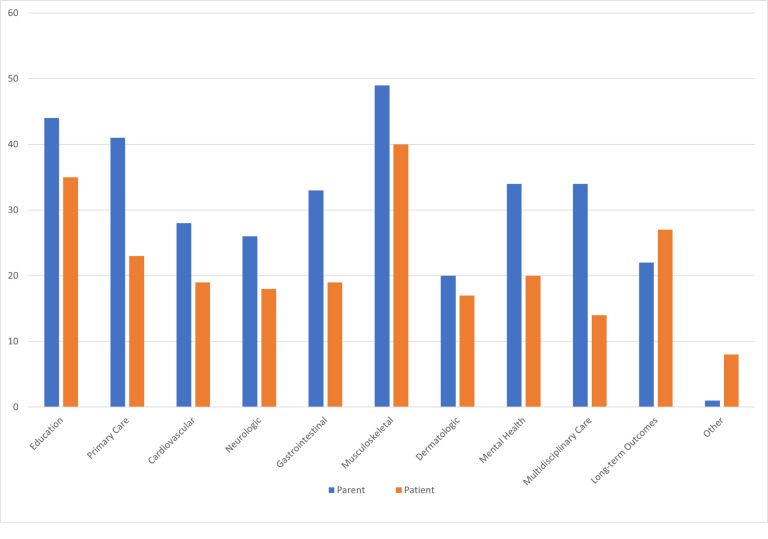
Concerns and Clinical Needs of Parent and Patient After Diagnosis of EDS

## Data Availability

The datasets generated and analyzed during the current study are available from the corresponding author on reasonable request.

## Abbreviations

EDS: Ehlers Danlos Syndrome
QoL: Quality of life
IRB: Institutional review board
